# Bad housekeeping: why do aphids leave their exuviae inside the colony?

**DOI:** 10.1186/1471-2148-8-338

**Published:** 2008-12-19

**Authors:** Frédéric B Muratori, David Damiens, Thierry Hance, Guy Boivin

**Affiliations:** 1 Unité d'écologie et biogéographie, Centre de recherche sur la biodiversité, Université catholique de Louvain, croix du sud, 4, 1348 Louvain-la-Neuve, Belgium; 2 Centre de Recherche et de Développement en Horticulture, Agriculture et Agroalimentaire Canada, Boul. Gouin, 430, Saint-Jean-sur-Richelieu, J3B 3E6 Québec, Canada; 3 Department of Natural Resource Sciences, McGill University, 21111 Lakeshore Road, Sainte-Anne-de-Bellevue, H9X 3V9 Québec, Canada

## Abstract

**Background:**

Animals can gain protection against predators and parasites by living in groups. The encounter-dilution effect provides protection when the probability of detection of a group does not increase in proportion to group size (i.e. encounter effect), so that predators do not offset the encounter effect by attacking more members of the group (i.e. dilution effect). In this paper, we propose a novel mechanism by which prey insects could gain by producing decoys that act as multiple targets for predators or parasitoids if these decoys are recognised as preys or hosts and negatively affect the patch foraging strategy of these predators and parasitoids. Such a decoy mechanism could be present in aphid colonies in which aphid exuviae are recognised and attacked by Aphidiine wasps.

**Results:**

We conducted a behavioural study to evaluate the effect of exuviae on parasitoid patch residence time and egg allocation in experimental aphid patches with or without exuviae. We showed that exuviae are recognised and attacked at the same level as aphids when both are present in the patch. While parasitism rate was not significantly lower in patches with exuviae when the parasitoid left the patch, the time wasted by parasitoids to handle exuviae did influence the patch residence time. As a consequence, the attack rate on the live aphids was lower in patches that contain exuviae.

**Conclusion:**

Aphids had more time available to flee and thus each individual might gain protection against parasitoids by leaving their exuviae near and within the colony. These results demonstrate that the encounter-dilution effect provided by living in a group can be enhanced by extra-materials that act as decoy for natural enemies.

## Background

Living gregariously is one of the defence mechanisms that animals have evolved against predators and parasitoids [1]. Groups can provide protection either through dilution of the attack risk, through collective aggressive and/or escape behaviour or through special structures (e.g. nests of some caterpillars) [2,3].

The major concept behind group protection is selfish herding [4]. Living in a group increases competition for food and mates but the fitness of each individual can nonetheless increase through the encounter-dilution effect [5]. The group provides protection when the probability of detection of the group does not increase in proportion to its size (i.e. encounter effect), and when the predator does not offset the encounter effect by attacking more members of the group (i.e. dilution effect). The dilution effect is itself influenced by the functional response of the predator. Experimental data on selfish herding are available for prey-predator relationships [5], but only a few studies have discussed host-parasitoid systems [6].

Most studies on the encounter-dilution effect took into account only the individuals forming the group [7] and little is known about the effect of the presence of either non-host species or host by-products in or near the group. The term "decoy effect" is used to designate situations where the host species can gain from living in a group with a non-host species that reduces the efficiency of a natural enemy [8-10]. The decoy-effect can thus modify the encounter-dilution mechanism [11,12]. Decoys could have an impact in a host-parasitoid system if they are recognised and attacked as hosts and therefore decrease the rate of encounter with healthy hosts. Such a "host decoy mechanism" might be present in aphid colonies. During the development of an aphid colony, the exuviae of aphids often remain within the colony for several days and oviposition behaviours towards aphid exuviae by Aphidiine wasps have been reported [13,14]. We propose here that exuviae can provide protection to the colony by acting as decoys causing a decrease in the patch foraging efficiency of parasitoid females.

Female parasitoids enter a host patch with an estimate, innate or learned, of the patch quality and this estimation is then refined as the parasitoid exploits the patch [15]. Mechanistic models of patch foraging strategies by insect parasitoids show that each oviposition has either an incremental or decremental effect on the patch allocation time of each parasitoid species [16-20]. Oviposition in a low quality host (e.g. a previously parasitised host) generally increases the patch-leaving tendency of the female while oviposition in a good quality host has the opposite effect. Theoretically, any encounter within the host patch, including encounters with host exuviae, could have an effect on the exploitation strategy of the female parasitoid.

However, for a parasitoid female, exuviae can also be used as reliable cues for the presence of aphid hosts [21]. Parasitoids usually show arrestment and intensive searching response around areas where host waste (such as honeydew) are encountered [22-26]. A correlation between number of exuviae and aphid density has been shown in *Sitobion avenae *colonies in natural conditions (Muratori and Hance, unpublished data) suggesting that exuviae could act as a reliable cue of aphid patch presence and quality. Aphids could thus be under two conflicting evolutionary pressures since the exuviae in a colony could incur costs from colony detection and benefits from a decoy effect.

In this paper we test the hypothesis that the presence of exuviae within a patch can afford protection to aphids by increasing the exploitation cost of parasitoids. Three predictions were tested: (i) parasitoids should perform attacks on exuviae even if aphids are present, (ii) if exuviae are perceived as low quality hosts, the parasitoid should leave the patch earlier in patches with exuviae (assuming that other patches in the habitat are percieved as better quality patches), (iii) aphids should be encountered at a lower rate in patches with exuviae (as predicted by the decoy effect hypothesis). We tested these predictions through an analysis of the behaviour of *Aphidius rhopalosiphi *De Stefani Perez (Hymenoptera: Aphidiinae) foraging on patches of the grain aphid, *Sitobion avenae *Fabricius (Homoptera: Aphididae), with or without exuviae.

## Results

### Behavioural events (Prediction 1)

Mean numbers of behavioural events noted under each condition are summarised in Table 1. The number of aphid encounters was higher when 16 aphids were present per patch than when 8 aphid were present ('16ap' vs '8ap'; F = 33.84; p < 0.0001). Moreover, aphids were not encountered less frequently when exuviae were present ('8ap + 8ex [ap]' vs '8ap'; F = 0.86; p = 0.359) and exuviae were encountered more frequently than aphids in the '8ap + 8ex' patch ('8ap + 8ex [ex]' vs '8ap + 8ex [ap]'; F = 4.82; p = 0.034). The total number of encounter events (exuviae plus aphids) was higher in patches with exuviae than in '8ap' patches ('8ap + 8ex [sum]' vs '8ap'; F = 61.60; p < 0.0001) and did not significantly differ from the number of encounters in '16ap' patches ('16ap' vs '8ap + 8ex [sum]'; F = 0.75; p = 0.393).

**Table 1:** Behavioural events towards aphids and exuviae.

	n	Enc	Ant	Ovip	PRT
8ap	17	9.3^a^ (0.8)	5.7^a^ (0.5)	7.2^a^ (0.6)	224.1^b^ (24.6)
8ap + 8ex [ap]	19	10.3^a^ (0.7)	6.7^a^ (0.6)	7.7^a^ (0.5)	509.8^a^ (78.3)
8ap + 8ex [ex]	19	13.4^b^ (1.2)	12.1^b^ (1.1)	8.1^a^ (0.7)	509.8^a^ (78.3)
8ap + 8ex [sum]	19	23.7^c^ (1.7)	18.8^c^ (1.5)	15.8^b^ (0.9)	509.8^a^ (78.3)
16ap	21	21.4^c^ (2.0)	10.9^b^ (1.3)	14.7^b^ (1.3)	560.7^a^ (71.1)

df		4	4	4	2
		F = 23.24	F = 23.84	F = 24.85	Chi2 = 29.96
p		<0.0001	<0.0001	<0.0001	<0.0001

Means (± SE) with different letters within the same column are significantly different (P < 0.05). 'Enc' = number of encounters, 'Ant' = number of antennal contacts, 'Ovip' = Number of ovipositor insertions, 'PRT' = patch residence time, '8ap' = 8 aphid patches, '8ap + 8ex' = 8 aphid and 8 exuviae patches, '16ap' = 16 aphid patches, '8ap + 8ex [ap]' = behaviour towards the aphids in the '8ap + 8ex' patches, ' 8ap + 8ex [ex]' = behaviour towards the exuviae in the '8ap + 8ex' patches, ' 8ap + 8ex [sum]' = behaviour toward both the aphids and the exuviae in the '8ap + 8ex' patches.

The number of antennal contacts on the aphids and exuviae followed the same trends except that when exuviae and aphids were both present, the parasitoid showed a higher number of antennal contacts on the exuviae than on the aphids ('8ap + 8ex [ex]' vs ' 8ap + 8ex [ap]'; F = 20.34; p < 0.0001).

The number of observed ovipositor insertions on aphids was higher in '16ap' patches than in other patches, but the total number of ovipositor insertions was not significantly different between the '16ap' patches and '8ap+8ex' patches (Table 1; '8ap + 8ex [sum]' vs '16ap'; F = 0.47; p = 0.498). When exuviae and aphids were present, they were stung with the same frequency (table 1; '8ap + 8ex [ap]' vs '8ap + 8ex [ex]', F = 0.14; p = 0.7103). The patch composition had no significant effect on the number of mummies produced (Chi^2 ^= 0.87, p = 0.645).

### Patch residence time (prediction 2)

**Figure 1:**
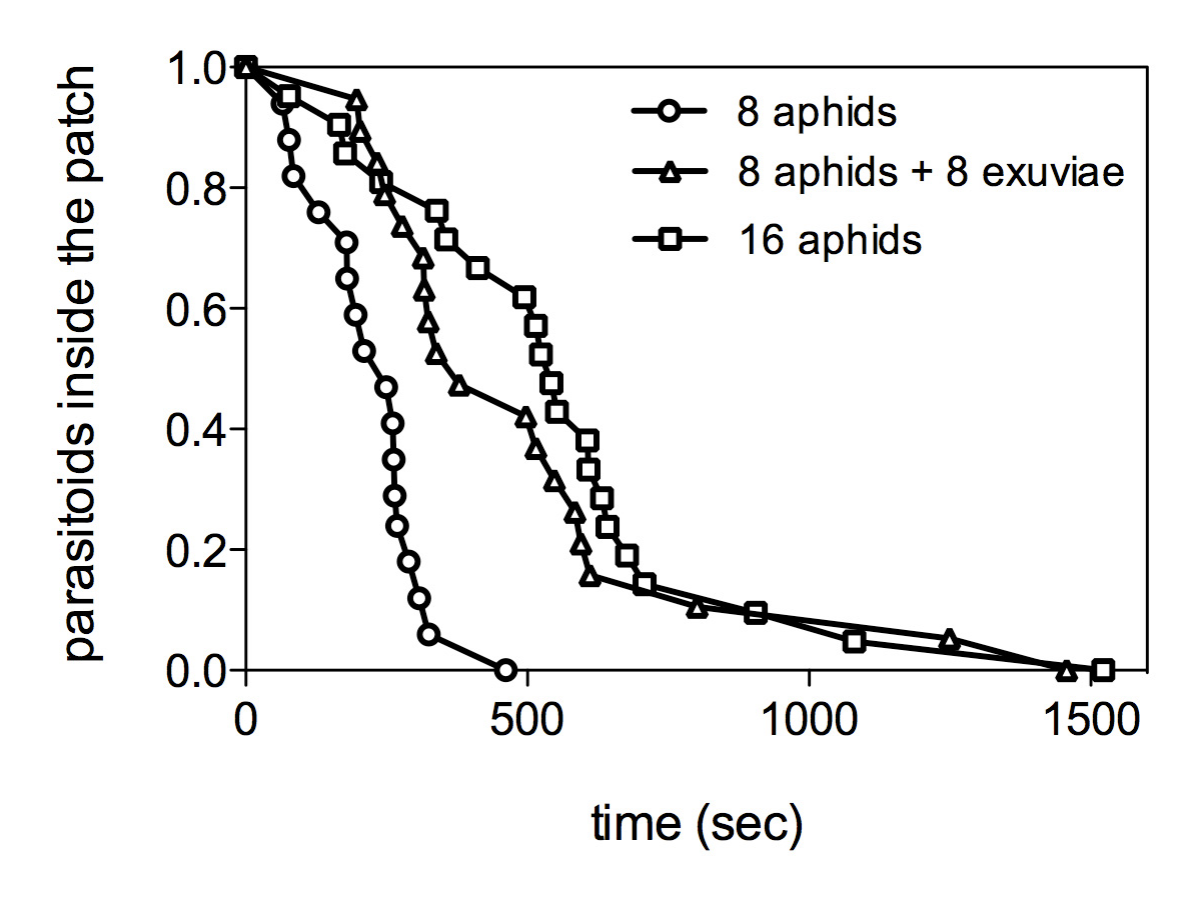
**Differential patch residence time according to patch composition**. The proportion of females remaining inside the patch according to time is lower when exuviae are present.

Patch residence time (PRT) was strongly influenced by patch composition (Log-rank test: Chi^2 ^= 29.96, df = 2, p < 0.0001) (Figure 1). Further simultaneous treatment comparisons showed that the density had an effect on the patch residence time ('8ap' vs '16ap': Chi^2 ^= 22.39; p < 0.0001) and that the presence of exuviae increased the patch residence time of females ('8ap' vs '8ap + 8ex': Chi^2 ^= 16.70; p < 0.0001). The time invested by parasitoids in patches with 16 aphids vs. 8 aphids + 8 exuviae was not significantly different (Chi^2 ^= 0.68; p = 0.408).

### Rate of behavioural events (prediction 3)

**Figure 2:**
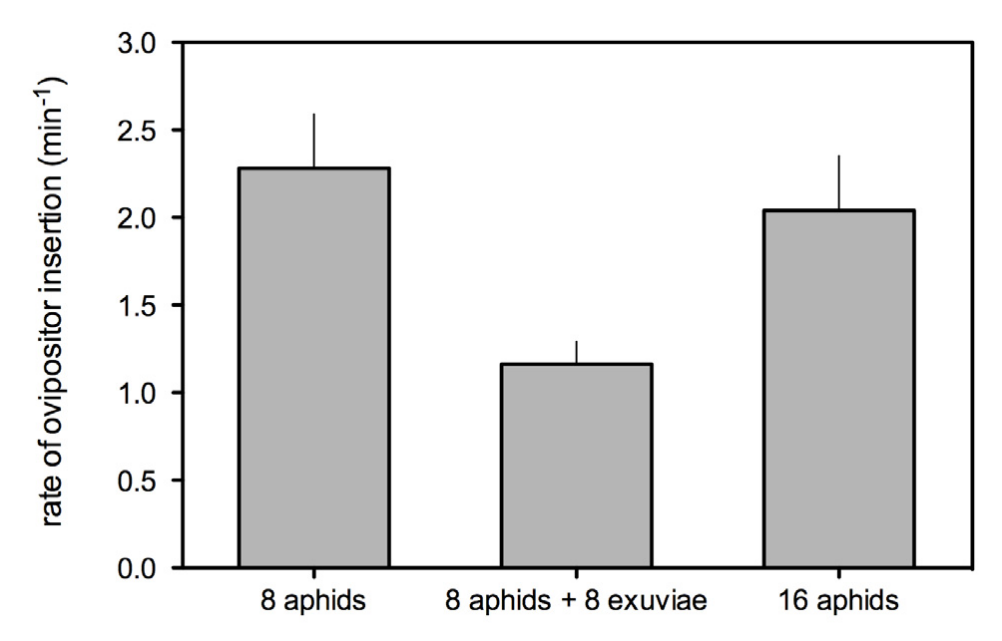
**Effect of patch composition on the rate of ovipositor insertion on aphids**. Error bars indicate standard errors. Means that share the same letter are not significantly different.

Despite the longer PRT in the '8ap + 8ex' patches, the number of ovipositor insertions was not significantly different between patches with/without exuviae ('8ap + 8ex' vs '8ap': F = 0.56; p = 0.4584). As a consequence, the rate of ovipositor insertion on aphids was significantly lower when both aphids and exuviae were present in the patch (F = 9.00; p = 0.0004) (Figure 2).

### Rate of ovipositor contacts curves

**Figure 3:**
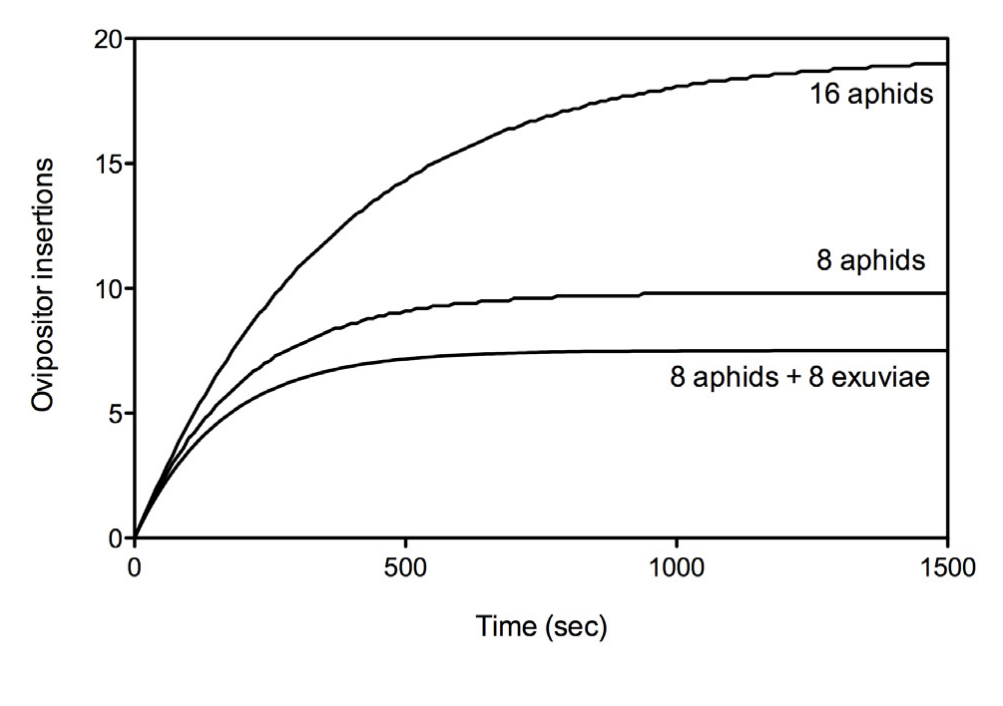
**Effect of patch composition on the modelled number of ovipositor insertions against time**.

Non-linear regression procedures gave the estimated parameters for equation 2 that were used to draw the curves of the modelled number of ovipositor insertions against time as a proxy for estimate the fitness curve for each treatment (Fig. 3). The longer a parasitoid stayed in a patch, the more it decreased the availability of healthy hosts thus decreasing the stinging rate. Figure 3 shows the fitness gain curve of a foraging female in each patch type. The curve that estimates the fitness gain obtained from a patch with exuviae is lower than the gain obtained from both patches without exuviae, but this trend is not significant (overlap of confidence intervals for the parameter Wmax and the parameter b between treatment '8ap' and '8ap + 8ex').

## Discussion

This study shows that extra-host material such as exuviae could play a role in group protection against parasitoids through a decoy mechanism. Aphids are under selection pressure (1) to reduce the amount of kairomone they produce and/or (2) to stay away from these traces by moving from them or by removing them. From this, it follows that when aphids leave their exuviae in their colony they face a trade-off between their effect as decoys that disturb parasitoid patch exploitation and their effect as cues used by parasitoid females to locate the aphid patch. It has been shown that many caterpillars that construct and inhabit leaf shelters ballistically eject their faecal pellets (frass) to great distances and at great speed to reduce the presence of chemical cues for natural enemies [27]. Aphids are also known to use their hind legs to throw honeydew droplets away from the colony [28]. Since aphid exuviae are covered by epicuticular waxes that have a kairomonal activity for the parasitoids [14], one would expect aphids to moult outside the colony or remove their exuviae. However, such behaviour has not been observed suggesting that either the gain from the decoy mechanism is higher than the risk of increased detection or that despite the cost of keeping exuviae, the behaviour of moulting away from the colony is not available in the range of behaviours on which selection can act.

We made three predictions based on the hypothesis that the presence of exuviae in the patch could influence the time and energy cost for the parasitoid and/or the parasitoid host patch time allocation. The first prediction was verified, since exuviae were recognised and attacked in a similar way as aphids. Our second prediction was that if exuviae were perceived as low quality hosts, the parasitoid should leave the patch earlier in patches with exuviae. In contrast, we found that parasitoid females spent more time in patches that contained exuviae ('8ap + 8ex') than in patches that contained only aphids ('8ap') suggesting that these females either did not recognise exuviae as low quality hosts or needed time to correctly identify them. Since our results showed that the number of parasitized aphids in the foraged colony was not significantly different when exuviae where present, the potential gain for the individuals comes from the time available to escape from the colony. Our experimental set-up (horizontal patch) did not allow aphids to perform the whole range of escaping behaviour, such as dropping from the plant [29]. This hypothesis should be tested in a more realistic situation with whole vertical plants.

Nevertheless, we found that the exuviae had a negative effect on the time allocation of the parasitoid. As predicted (third prediction), our results showed that the presence of exuviae increased the patch residence time of female parasitoids. Due to variability between females, the parameters of the fitness gain models fitted to the data (number of ovipositor insertions against time) were not significantly different between the '8ap' and '8ap + 8ex' patches (Fig. 3), but the rate of ovipositor insertions was significantly affected by the presence of exuviae (Fig. 2).

For aphids, keeping exuviae around the colony appears to be potentially advantageous as female parasitoids invested time manipulating exuviae, leading to an increase of their patch residence time. We did not investigate here the mechanisms of patch leaving rules that lead to an increase in patch residence time when exuviae are present and modifications in aphid defense could be involved. Having female parasitoids investing more time in a colony could be advantageous for aphids if that increased patch visit duration enables aphids to (i) respond to the alarm pheromone contained in the cornicle secretion emitted when aphids are under parasitoid attack [30,31] and/or (ii) escape from the colony [32]. Under a more natural situation, the aphid would be free to drop from the plant as an escape behaviour [33]. Moreover, it is possible that direct defensive aphid reactions, such as legs kicking or abdomen movement, might be more frequent in patches without exuviae as the rate of encounters with aphids is higher in these patches. When exuviae are present, a lower rate of encounters with aphids might lead to an increase in patch leaving tendency of the parasitoid [19,34,35], thus increasing the decoy effect of the exuviae. More detailed experimental setup allowing the study of mechanistic models are needed to disentangle the effect of events such as aphid defensive behaviours.

The reduced efficacy of female parasitoids within a patch containing exuviae could also benefit related aphids of neighbouring patches. When a parasitoid female increases its patch residence time in one colony, the probability that surrounding colonies will be found and parasitised by this female decreases. Thus, if the relatedness of two aphid colonies is high, kin selection could favour the occurrence of a decoy mechanism. In nature, the genetic diversity of aphid populations is usually low within a micro-habitat, as only a few parthenogenetic females will give rise to a multiclonal population with low genetic diversity [36,37], but the relatedness of nearby colonies in the same micro-habitat has still to be evaluated.

For female parasitoids, the presence of exuviae is costly since they spent time handling exuviae, performing not only antennal contacts but also ovipositor insertions. In Aphidiine wasps, the ovipositor valvulae III, that contact the host cuticle during penetration of the ovipositor tip, are covered by both mechano- and chemo-receptors that receive information from the host cuticle [38-40]. Additional information through ovipositor contact is needed and, as a consequence, the parasitoid spent more time handling exuviae. As a result, it took the females longer to reach the same level of parasitism in patches that contained both aphids and exuviae. Moreover, our results suggest that the parasitoid did not exhibit learning that could reduce the handling time of subsequent encounters of exuviae by reducing the frequency of ovipositor insertion [41,42]. When females invest more time in patches with exuviae they risk (i) an increase in aphid defensive behaviours, (ii) an increase of patch detection by hyperparasitoids or competitors and (iii) the reduction of the time left to locate other patches. The latter depends on the number of patches a parasitoid is expected to find during its lifespan. It would be interesting to investigate if the cumulated time costs associated with exuviae presence have an impact on the lifetime fitness of the females and if the response of females changes with their life expectancy [20].

In the present study, aphids and exuviae were alternately distributed on the experimental patches while in nature the exuviae are more likely to be found around the colony. Such a distribution of exuviae could magnify the effect shown here. The architecture of the host-plant could also influence the distribution of the exuviae as they are likely to be trapped by parts of the plant that are situated under the aphid colonies. Exuviae could thus be seen as increasing the complexity of the micro-habitat around the aphid, a factor known to reduce the searching efficiency of parasitoids [43,44].

## Conclusion

Here we present a new aspect of the decoy effect hypothesis that could have led to the selection of "exuviae keeping" within aphid colonies as a protection against parasitoids. This shows that group protection analysis should be extended to extra-material produced by a prey individual, in addition to the individual itself.

## Methods

### Insect rearing

*Aphidius rhopalosiphi *was reared on *S. avenae *maintained on winter wheat (*Triticum aestivum *cv "Corvus") at 19.5 ± 0.6°C, 40–50% relative humidity and 16 h L : 8 h D photoperiod. Parasitoids were synchronised on L2 aphid cohorts. In a 50 × 50 × 30 cm framed wooden cage with fine mesh on each side, pairs of parasitoids (1 pair per 50 aphids) were released for 24 h. Ten days later, mummies were gently removed from the wheat leaves with a scalpel blade and kept in Petri dishes in groups of 50. Females used in all bioassays were 2 days old, mated (isolated with two males for 24 h), fed (honey:water 50:50) and experienced (placed for 1 h with 10 aphids). Females were isolated in a Petri dish with food for 24 h before trials.

### Behavioural bioassay

In a 15 cm diameter glass Petri dish, 16 pieces of wheat leaf (separated by 0.5 cm) were placed within a 9 cm diameter circle drawn with red ink that was considered the border of the experimental patch. Three types of patches were set up: (1) 16 aphids with one aphid/leaf ('16ap'), (2) 8 aphids and 8 exuviae equally distributed to form a latin square ('8ap + 8ex') and (3) 8 aphids distributed every other piece of leaf ('8ap'). The experimental arena was placed on a light table (2500 LUX) in a dark room (22 ± 1°C). One female parasitoid was released at the centre of a patch. The number of aphid and exuviae encounters, antennal contacts and ovipositor insertions as well as the patch residence time were recorded using event recorder software The Observer^® ^5.0 (Noldus Technology, Wageningen). A wasp was considered to leave the patch when it remained outside the red limit of the patch for more than 60 sec. treatment '8ap') was conducted with a new female, a clean Petri dish (alcohol and distilled water washed), and fresh wheat leaves.

### Parasitism rate

Once the female left the patch, all aphids were transferred and reared on an artificial diet [45] under controlled conditions (19.1 ± 1.0°C, 60% R.H) for 14 days. Aphids were checked daily for mummification. In order to include aphid mortality, the parasitism rate was computed as the [number of mummies/(number of still living aphids + number of mummies)].

### Statistical analysis

Patch residence times in the three types of patches were compared using a log-rank test (PROC LIFETEST). Log-linear regressions (PROC GENMOD) were used to analyse the effect of the treatments on the number of behavioural events. The number of ovipositor insertions per unit of time (i.e. the rate of ovipositor insertions) was compared between the 3 treatments with an analysis of variance (PROC GLM). Parasitism rates in patches were compared by a logistic regression (PROC GENMOD). All analyses were done with SAS/STAT^® ^statistical software (SAS Institute Inc, Cary, North Carolina). In order to evaluate the fitness gain (approximated from the number of aphids stung) acquired by the parasitoid during the patch visit, non-linear regressions were produced using PROC NLIN [46]. This iterative procedure was used to find the least square estimates of the parameters of the fitness gain (W) equation [47-49]:

W(t) = Wmax [1 - exp(- b t)]

where b is the rate at which W raises to its asymptote, Wmax, and t is the time spent in the patch.

## Authors' contributions

FBM designed, conducted and analysed the lab research and prepared the manuscript. DD contributed to the manuscript. TH assisted in planning the research and contributed to the manuscript. GB contributed to the design of the research and contributed to the manuscript. All authors approved the manuscript.
